# Low seroprevalence of SARS-CoV-2 infection among healthcare workers of the largest children hospital in Milan during the pandemic wave

**DOI:** 10.1017/ice.2020.401

**Published:** 2020-08-06

**Authors:** Antonella Amendola, Elisabetta Tanzi, Laura Folgori, Lucia Barcellini, Silvia Bianchi, Maria Gori, Giulia Cammi, Elena Albani, Gian Vincenzo Zuccotti

**Affiliations:** 1 Department of Biomedical Sciences for Health, University of Milan, Milan, Italy; 2 Coordinated Research Centre “EpiSoMI,” University of Milan, Milan, Italy; 3 Department of Paediatrics, Children Hospital V. Buzzi, University of Milan, Milan, Italy

Lombardy, Northern Italy, was the first region within a Western country to be severely hit by the spread of severe acute respiratory coronavirus virus 2 (SARS-CoV-2). The coronavirus disease 2019 (COVID-19) pandemic started officially in Italy on February 21, 2020,^1^ although today it is recognized that the virus had been circulating unnoticed for at least a month prior to that date.^2,3^ Several nosocomial outbreaks occurred in the first phase of the epidemic, and healthcare workers (HCWs) were the most vulnerable cohort for COVID-19 due to frequent and close contact with COVID-19 patients without, at least in an initial phase, adhering to strict hygienic measures.

We conducted a cross-sectional seroprevalence study among the HCWs of the largest pediatric hospital in Milan during the period of maximum epidemic activity, when Lombardy accounted for 37% of cases and 53% of deaths in the country.^4^

## Methods

We analyzed serum samples collected on April 15, 2020, from 663 workers (108 males and 555 females; median age, 44 years) at the Buzzi Hospital in Milan, where the first confirmed COVID-19 pediatric patient was hospitalized on February 28 and, until the time of this study, where 40 COVID-19 cases were managed. All HCWs and non-HCWs who decided to take part in the survey were interviewed to review potential occupational exposures to COVID-19 patients, symptoms, and use of personal protective equipment (PPE) as recommended by the WHO.^5^

Of 742 employees, 547 HCWs and 116 non-HCWs (ie, biologists, pharmacists, laboratory technicians, administrative employers) were included in this study. All had no symptoms of COVID-19 at the time of blood collection. Approximately 41% of subjects reported symptoms during the weeks preceding sampling, but none had been hospitalized or had undergone nasopharyngeal swab for the detection of SARS-CoV-2 RNA. For 304 of the 547 HCWs (55.6%), at least 1 contact with confirmed COVID-19 patients was known.

Anti–SARS-CoV-2 IgG antibodies were detected using a semiquantitative enzyme-linked immunosorbent assay (ELISA) (Euroimmun Medizinische Labordiagnostika, Lubeck, Germany) according to the manufacturer’s instructions.

Comparisons between subject characteristics, work settings and SARS-CoV-2 IgG positivity were made using the χ^2^ test. *P* < .05 was considered statistically significant (2-tailed test). All statistical analyses were performed using OpenEpi version 3.03a software.

## Results

Overall, 34 subjects tested positive for SARS-CoV-2 IgG, with a prevalence of 5.13%; most of these (26 of 34, 76.5%) reported symptoms related to COVID-19, mostly during the month of March. Seroprevalence was almost identical among HCWs and non-HCWs (5.12% vs 5.17%, respectively; *P* = .95), but the rate was significantly higher among males compared to females (9.26% vs 4.32%; *P* = .049).

Two wards, surgery and pediatric intensive care, showed a significantly higher frequency of infection than the others (22.2% vs 4.4%, *P* < .001 and 14.3% vs 4.5%, *P* < .01, respectively). Table 1 shows the percentage of anti–SARS-CoV-2 IgG positivity broken down by HCWs characteristics and hospital wards.

**Table 1.** Subjects Characteristics, Work Settings, and SARS-CoV-2 IgG Positivity

Characteristic	SARS-CoV-2 IgG+No. (%)
Female (N = 555)	24 (4.32)^a^
Male (N = 108)	10 (9.26)^a^
Total (N = 663)	34 (5.13)
**Profession**	
Healthcare worker (N = 547)	28 (5.12)
Physician (N = 214)	10 (4.67)
Nurse (N = 216)	13 (6.02)
Other health technicians (N = 117)	5 (4.27)
Non-healthcare worker (N = 116)^b^	6 (5.17)
**Setting**	
Specialist outpatient services (N = 63)	4 (6.34)
Surgery (N = 27)	6 (22.22)^c^
Pediatric (N = 80)	1 (1.25)
Pediatric emergency room (N = 55)	1 (1.82)
Neonatal intensive care (N = 47)	1 (2.13)
Pediatric intensive care (N = 42)	6 (14.29)^d^
Pre- and postnatal (N = 181)	6 (3.31)
Administration/Pharmacy/Laboratory (N = 70)	6 (8.57)
Others (N = 98)	3 (3.06)

a Female vs male: 4.32% vs 9.26%, *P* < .05.

b Biologists, pharmacists, laboratory technicians and administrative employers.

c Surgery vs all the others: 22.2% vs 4.4%, *P* < .001.

d Pediatric intensive care vs all other wards: 14.3% vs 4.5%, *P* < .01.

Among HCWs, the percentage of seroconversion was 6.58% (20 of 304) and 3.29% (8 of 243) in those with or without contact with confirmed COVID-19 patients, respectively (*P* = .08). The seroconversion rate was significantly higher among HCWs with PPE-free contact than those who had worn PPE: 21.6% (8 of 37) versus 4.5% (12 of 267) (*P* < .01). All IgG positive HCWs with symptoms had had contact with COVID-19 patients, without PPE, in the first 2 weeks of March. Of the 7 asymptomatic HCWs who were positive for IgG, 5 had no known contact with confirmed COVID-19 patients, and 2 reported PPE-protected contact.

## Discussion

To date, serological data are lacking and the actual spread of the infection remains undetermined, particularly among HCWs who manage the COVID-19 emergency in a territorial or hospital setting. We examined HCWs of the largest pediatric hospital in Milan to determine SARS-CoV-2 seroprevalence in an area with high epidemic density.

Overall, we found a IgG prevalence of ~5% on April 15,^4^ when Lombardy had counted 62,153 confirmed cases and 11,377 deaths. Interestingly, healthcare professionals and other hospital employees showed the same percentage of IgG positivity (5.13% vs 5.17%). Notably, this percentage is completely comparable (5%) to that found in the first week of April in a recent study conducted on blood donors from the same geographical area (Milan).^6^ Among HCWs who became infected, the highest risk factor was having contact with COVID-19 patients during the very early stages of the pandemic, when the availability of PPE was still inadequate. Among HCWs who did not have contact with confirmed cases, the percentage of infection was low (3.29%), even lower (although not significantly) than among non-HCWs (5.17%). Serological analysis indicated that 25% of infected HCWs were asymptomatic with no contact with confirmed COVID-19 patients (71.4%) or had PPE-protected contact (28.6%). A limitation to this study could be the lack of information regarding staff-to-staff transmission and potential community-associated risks.

In conclusion, our data indicate that the prevalence of SARS-CoV-2 among pediatric HCWs is low and similar to community prevalence, suggesting that there is no increased risk within hospitals providing appropriate PPE.

These results are of particular relevance considering that this area was among those with the highest epidemic density worldwide and that the virus had already spread unnoticed since mid-January 2020. The hypothesis of a minor role of children in the spread and transmission of SARS-CoV-2^7^ should be explored. Further retrospective serological investigations among children with respiratory symptoms that were hospitalized or had access to the emergency room before the official start of the COVID-19 outbreak in Italy will allow to date the introduction of the virus in the pediatric population.
